# The gender gap and healthcare: associations between gender roles and factors affecting healthcare access in Central Malawi, June–August 2017

**DOI:** 10.1186/s13690-020-00497-w

**Published:** 2020-11-17

**Authors:** Amee D. Azad, Anthony G. Charles, Qian Ding, Amber W. Trickey, Sherry M. Wren

**Affiliations:** 1 Stanford University School of Medicine, 291 Campus Drive, Stanford, CA 94305 USA; 2 University of North Carolina Department of Surgery, Chapel Hill, NC USA; 3 Stanford-Surgery Policy Improvement Research & Education Center, Stanford, CA USA; 4 Palo Alto Veterans Healthcare System, Palo Alto, CA USA

**Keywords:** Gender, Inequality, Disparity, Gender role, Access, Healthcare, Empowerment

## Abstract

**Background:**

Women in low and middle-income countries (LMICs) do not have equal access to resources, such as education, employment, or healthcare compared to men. We sought to explore health disparities and associations between gender prioritization, sociocultural factors, and household decision-making in Central Malawi.

**Methods:**

From June–August 2017, a cross-sectional study with 200 participants was conducted in Central Malawi. We evaluated respondents’ access to care, prioritization within households, decision-making power, and gender equity which was measured using the Gender-Equitable Men (GEM) scale. Relationships between these outcomes and sociodemographic factors were analyzed using multivariable mixed-effect logistic regression.

**Results:**

We found that women were less likely than men to secure community-sourced healthcare financial aid (68.6% vs. 88.8%, *p* < 0.001) and more likely to underutilize necessary healthcare (37.2% vs. 22.4%, *p* = 0.02). Both men and women revealed low GEM scores, indicating adherence to traditional gender norms, though women were significantly less equitable (W:16.77 vs. M:17.65, *p* = 0.03). Being a woman (Odds Ratio (OR) 0.41, 95% confidence interval (CI) 0.21–0.78) and prioritizing a woman as a decision-maker for large purchases (OR 0.38, CI 0.15–0.93) were independently associated with a lower likelihood of prioritizing women for medical treatment and being a member of the Chewa tribal group (OR 3.87, CI 1.83–8.18) and prioritizing women for education (OR 4.13, CI 2.13–8.01) was associated with a higher odds.

**Conclusion:**

Women report greater barriers to healthcare and adhere to more traditional gender roles than men in this Central Malawian population. Women contribute to their own gender’s barriers to care and economic empowerment alone is not enough to correct for these socially constructed roles. We found that education and matriarchal societies may protect against gender disparities. Overall, internal and external gender discrimination contribute to a woman’s disproportionate lack of access to care.

**Supplementary Information:**

The online version contains supplementary material available at 10.1186/s13690-020-00497-w.

## Background

Worldwide, women are disproportionately affected by economic vulnerability, lower social status, and limited access to education compared to men [1]. The importance of addressing gender inequalities in access to healthcare has been well-established in the literature with demonstrated reduction in mortality and morbidity for men and women alike [2]. The 2019 Lancet series on Gender Equality, Norms, and Health, 2015 Lancet Women and Health Commission, 2009 WHO Women and Health Report, and similar studies highlight how traditional gender norms that focus on women as caregivers and men as providers can impact health [3–5]. In these subordinate positions, women have limited decision-making power, access to resources, and have less economic and social utility than men [6, 7]. The expanding literature on the effects of gender discrimination demands a holistic view of women’s health – one that considers the obstacles women face throughout their lifetime and the compounding effects of how gender norms, roles, and relationships affect their health.

Gender disparities have a negative effect on women’s health that extends beyond biology alone [8, 9]. From female infanticide to “benign neglect” of girls under the age of 5 leading to poor nutrition and delays in seeking care to lower rates of seeking appropriate and early care in women, there are clear social, cultural, and structural practices that disadvantage girls and women [10]. One structural example of structural gender disparities can be found in the rates of hospital admissions. In high-income countries, the gender ratio for overnight hospital stays and emergency medical admissions is nearly equivalent [11–14]. Women in low- and middle-income countries (LMICs) present less frequently in similar medical circumstances, with studies reporting male-to-female ratios of 2.2 for emergency abdominal surgeries and 1.4 for general admissions, excluding obstetric care [15, 16]. These studies have determined that in circumstances where disease prevalence is equal among men and women, gender roles, socially constructed beliefs and attitudes towards specific genders, may contribute to inequalities in health [17–19].

Traditionally measured barriers of access to care, such as distance from health facilities and the base cost of healthcare access, are generally found to affect both genders in similar ways [20, 21]. Sociocultural determinants such as gender norms, autonomy, and decision-making power have been proposed as potential barriers to access that disproportionately affect women, but rigorous research on these effects are limited in scope [22–25]. In studies of maternal or child healthcare utilization, higher social status, education level, and increased financial autonomy of women predict higher rates of prenatal healthcare utilization [26, 27]. This finding reveals a crucial connection between economic and social empowerment of women and health in the literature that has not yet extended to the full life course of women beyond their reproductive years [28]. Therefore, this study was designed to: 1) assess the sex ratio in hospital admissions in the referral hospital in Lilongwe, Malawi and 2) conduct a community-based survey to explore societal beliefs towards gender roles and the choices of individuals on the basis of these factors that may undermine women’s health.

## Methods

### Study setting

Malawi is a low-income country of 17.2 million people in South-East Africa with a United Nations Development Programme Gender Development Index (GDI) score of 0.921, placing it well below gender parity. Malawi has a three-tiered national healthcare service network of health centers, district, and central or tertiary hospitals that is free to all Malawians at the point of delivery. The primary study site, Kamuzu Central Hospital (KCH) in the capital of Lilongwe, Malawi, is a tertiary care center, accepting referrals from 8 district hospitals in the central region of Malawi. Hospital bed capacity is 780 and it serves a catchment population of approximately 5 million people with 59 physicians and 286 nursing staff members. Two local markets (Msundwe and Mitundu Markets), located within a 40 km perimeter of Lilongwe, were included as secondary study sites.

### Census procedures

Over a 6-week period from June 23, 2017 to August 7, 2017, a daily census was collected at the study hospital. Data were collected by one researcher (AA) for all patients admitted to KCH non-surgical, non-obstetric/gynecologic pediatric and adult medical wards. These wards were selected to represent conditions that has an equal distribution between genders because the prevalence of infectious and non-communicable diseases is more gender neutral than many surgical and all obstetric/gynecologic conditions [29]. Collected data elements included patient age, sex, primary diagnosis, and admitting ward. The researcher then entered the data into a REDCap electronic database at Stanford University for analysis [30].

### Survey procedures

Respondents were recruited using stratified systematic sampling by place and gender and were interviewed using a 6-domain tool adapted from surveys previously validated in international studies (Table 1) [31]. The Gender-Equitable Men (GEM) Scale was included in the community survey to capture gender attitudes regarding norms, roles, and equity [32]. Lower GEM scores indicate more traditional, less equitable gender role attitudes [33].

**Table 1:** Measures of Correlates of Gender Disparity in Lilongwe, Malawi, June–August 2017

**Demographic information**	
• Sex • Age • Tribal group (for analyses, this factor was categorized as Chewa vs Other) • District (for analyses, this factor was categorized as Lilongwe vs Other) • Religion (for analyses, this factor was categorized as Christianity vs Other) • Marital status • Employment status *(for analyses, this factor was categorized as employed vs unemployed) • Education level **(for analyses, this factor was categorized as low (primary and lower) vs high (secondary and higher)) • Income • Transport time • Spouse age • Spouse employment status* • Spouse education level** • Spouse income • Number of children in household • Number of people in household	
**Respondents’ gender attitude**	
• Measured using the Gender-Equitable Men (GEM) Scale from the International Men and Gender Equality Survey (IMAGES) (48) • For analyses, scores were categorized into high vs low gender equity	
**Respondent’s access to and utilization of healthcare**	
• Questions were from previous international studies(15, 49) • If you could not afford healthcare, could you get financial support from your family or community? • Have you ever been seriously ill and chosen not to seek healthcare?	
**For respondents’ prioritization of household members for medical treatment**	
• Questions were from previous international studies(50) • Whose would you prioritize for access to medical care first? • Measures were categorized as self, spouse, son, or daughter	
**Respondents’ healthcare decision-making power**	
• Assessed by use of the Demographic and Health Surveys (DHS) on Women’s Status and Empowerment • Who usually makes decisions about healthcare for yourself? • Who usually makes decisions about your children’s healthcare? • Who usually makes decisions about paying for your healthcare?	
**Respondents’ economic decision-making power**	
• Assessed by use of the Demographic and Health Surveys (DHS) on Women’s Status and Empowerment • Who usually makes decisions about making major household purchases (e.g. bicycles or land)? • Who usually makes decisions about minor household purchases (e.g. food and clothing)? • Who usually decides how the money you earn will be used?	

The survey was first pilot tested with 10 English-speaking respondents in the United States and 10 Chichewa-speaking respondents in Malawi and revised for clarity and timing prior to administration to the study population. Once finalized, the survey questions were administered orally in each respondent’s preferred language (predominantly Chichewa – a language spoken by 65–80% of Malawians [34]) by local research assistants in a single encounter. Study data were collected and managed using REDCap [30]. For quality assurance, two research assistants (JN and CC) administered all surveys and one research fellow (AA) supervised all survey administration.

Respondents at KCH were selected by requesting participation of every 5th adult appearing community member visiting the genitourinary, gastrointestinal, pediatric, endoscopy, or biopsy weekly clinics. Two market populations were also randomly sampled to reduce selection bias associated with the hospital sample. Market respondents were randomly selected by requesting participation of every 10th adult community member who walked through the main entrance of the marketplace. During oral interviews, respondents provided information on self and spouse demographics, gender equity attitudes, social prioritization, decision-making power, and barriers to care during the 20 min survey.

### Analysis

Sociodemographic characteristics included age, gender, marital status, education level, income, number of children, spouse age, spouse education level, spouse income. For analysis, we dichotomized prioritization and decision-making item responses into a preference for women vs. men (e.g. responses “son” and “father” were classified as a preference for men, along with “self” among respondents identifying as men). The GEM score dependent variable ranges from 1 to 24 and was dichotomized with a median divide: 1–16 indicating low equity and 17–24 indicating high equity [35].

Summary statistics are reported for sociodemographic characteristics. Descriptive statistics include frequency counts and percentages, mean and standard deviation for normally distributed variables, and median and interquartile range for non-normally distributed variables. Relationships between sociodemographic characteristics and response variables were assessed with chi-square or Fisher’s exact test for categorical variables. Continuous variables were compared by independent sample t-tests or Wilcoxon-Mann-Whitney test as appropriate. Hospital census admissions data were compared by patient gender and age group using chi-square tests.

To evaluate gender differences and sociodemographic characteristics associated with healthcare decision-making and economic prioritization, simple and multiple logistic regression analyses were performed. Respondents with missing values for dependent or independent variables were excluded from regression analysis. Categories with low frequencies (*n* < 10) were collapsed to create larger groups for regression analysis. Covariates associated with dependent variables at the level of *p* < 0.1 in simple logistic regression were considered in multiple regression models. We observed differing characteristics among respondents surveyed in the two study locations (hospital and market). Therefore, we utilized mixed effect logistic regression including random intercepts by survey location to account for clustering of individuals at each location. Variables were removed with stepwise backward selection until only variables associated with statistically significant dependent variables (*p* < 0.05) remained in final models. Statistical analyses were conducted using SAS v. 9.4 (Cary, NC). Statistical significance was indicated by two-sided *p* < 0.05.

## Results

### Sex disparity at Kamuzu central hospital (KCH)

During the hospital census, a total of 758 patients were admitted between June 23, 2017 and August 7, 2017. Obstetric/gynecologic and surgical admissions were not included in this census. Males comprised a higher proportion of total admissions (54.5% of medical admissions vs. females 45.5%, *p* = 0.01) with an M:F ratio of 1.2. Young females made up 44.3% of the pediatric ward, patients 16 and younger, and older females made up 47.8% of the adult wards (*p* = 0.07).

### Survey population demographics

Two hundred community members were surveyed, including 102 (51%) women and 98 (49%) men. Among these, 40 (20%) were in the market cohort and 160 (80%) the hospital cohort. There were significant differences between participant characteristics in the market and hospital cohorts, specifically for tribal group, marital status, spouse employment, age, spouse age, and transport time (Supplementary Table A). Overall, respondents reported earning a median 120,000 kwacha ($156.89) annually and supporting an average of 4 children. The survey population was largely married (82.5%), Protestant (58.5%), self-employed (89.0%), and with limited education (72.5% completed primary school or less). There were no significant differences in age, home region, tribal group, religion, or income by gender. Women were less likely to be married (*p* < 0.001), more likely to be self-employed (p < 0.001) and report lower education levels (*p* = 0.001) than men. Age and education of spouses were significantly different: with men reporting younger (mean age (years), Women (W): 45.9 vs. Men (M): 37.0 years, p < 0.001) and less educated spouses (primary school or less, W: 68.3% vs. M: 88.5%, p < 0. 001) than women (Table 2).

**Table 2:** Sociodemographic Characteristics of Participants by Gender in Lilongwe, Malawi, June–August 2017

	Total (***N*** = 200)	Women (***N*** = 102)	Men (***N*** = 98)	*P*-value*
**Region, n (%)**				1.00
Central Malawi	176 (88.0)	90 (88.2)	86 (87.8)	
Other	24 (22.0)	12 (11.8)	12 (12.2)	
**Tribal Group, n (%)**				0.41
Chewa	137 (68.5)	74 (72.5)	63 (64.3)	
Ngoni	38 (19.0)	16 (15.7)	22 (22.4)	
Other	25 (12.5)	12 (11.8)	13 (13.3)	
**Religion, n (%)**				0.72
Protestant	117 (58.5)	60 (58.8)	57 (58.2)	
Roman Catholicism	46 (23.0)	26 (25.5)	20 (20.4)	
Traditional Tribal	25 (12.5)	12 (11.8)	13 (13.3)	
Islam	9 (4.5)	3 (2.9)	6 (6.1)	
Other	3 (1.5)	1 (1.0)	2 (2.0)	
**Marital Status, n (%)**				< 0.001
Married	165 (82.5)	78 (76.5)	87 (88.9)	
Separated	13 (6.5)	10 (9.8)	3 (3.0)	
Widowed	10 (5.0)	10 (9.8)	0 (0.0)	
Single	12 (6.0)	4 (3.9)	8 (8.2)	
**Employment Status, n (%)**				< 0.001
Self-employed/Business	178 (89.0)	94 (92.2)	84 (85.7)	
Employed	12 (6.0)	1 (1.0)	11 (11.2)	
Unemployed	2 (1.0)	2 (2.0)	0 (0.0)	
Never Worked	6 (3.0)	5 (4.9)	1 (1.0)	
Other	2 (1.0)	0 (0.0)	2 (2.0)	
**Education Level, n (%)**				0.001
None	22 (11.0)	17 (16.7)	5 (5.1)	
Primary School	123 (61.5)	67 (65.7)	56 (57.1)	
Secondary School	52 (26.0)	17 (16.7)	35 (35.7)	
Higher Education	3 (1.5)	1 (1.0)	2 (2.0)	
**Spouse Employment, n (%)**				0.002
Self-employed/Business	148 (89.2)	67 (84.8)	81 (93.1)	
Employed	9 (5.4)	9 (11.4)	0 (0.0)	
Unemployed	4 (2.4)	1 (1.3)	3 (3.4)	
Never Worked	4 (2.4)	1 (1.3)	3 (3.4)	
Other	1 (0.6)	1 (1.3)	0 (0.0)	
**Spouse Education Level, n (%)**				< 0.001
None	16 (9.6)	2 (2.5)	14 (16.1)	
Primary School	115 (69.3)	52 (65.8)	63 (72.4)	
Secondary School	34 (20.5)	24 (30.4)	10 (11.5)	
Higher Education	1 (0.6)	1 (1.3)	0 (0.0)	
**Age, year, Mean (SD)**	41.7 (12.1)	41.4 (12.2)	42.0 (12.1)	0.74
**Spouse Age, year, Mean (SD)**	41.3 (12.4)	45.9 (12.4)	37.0 (10.9)	< 0.001
**Transport Time, hour, Median (IQR)**	3.0 (2.0, 6.0)	3.0 (2.0, 6.0)	3.0 (2.0, 6.0)	0.82
**Income (MWK)**^a^**, Median (IQR)**	120,000 (60,000, 300,000)	114,000 (60,000, 189,000)	180,000 (84,250, 480,000)	0.24
**Spouse’s Income (MWK)**^a^**, Median (IQR)**	125,000 (60,000, 300,000)	140,000 (80,000, 360,000)	122,500 (58,750, 300,000)	0.75
**Number of Children, Mean (SD)**	4.1 (2.1)	4.3 (2.0)	3.9 (2.2)	0.15
**Number in Household, Mean (SD)**	5.7 (2.0)	5.7 (1.7)	5.6 (2.2)	0.69
**GEM Raw Score, Mean (SD)**	17.2 (2.9)	16.8 (2.8)	17.7 (2.9)	0.03
**GEM Score Level, n (%)**				0.004
High	71 (35.5)	26 (25.5)	45 (45.9)	
Low	129 (64.5)	76 (74.5)	53 (54.1)	

Missing values: Spouse Employment = 34, Spouse Education = 34

*P-values were calculated by chi-square test, Fisher’s exact test, independent sample t-test, or Wilcoxon-Mann-Whitney test

^a^MWK indicates Malawian kwacha (1 US Dollar = 765.29 MWK as of July 16, 2019)

### Gender-equitable men (GEM) scale

Both men and women scored low on the GEM Scale (mean 17.21 of a possible 24) indicating adherence to traditional gender norms and lower gender equity. Women had lower GEM scale scores (16.77 vs. 17.65, *p* = 0.03), denoting more traditional, less equitable gender attitudes than men.

In regression analysis, factors that were independently associated with gender norm perspectives include respondent’s gender, travel time to Lilongwe, and number of children in the household. Being a woman was an independent predictor of a low GEM score (odds ratio, OR 0.39, 95% confidence interval, CI 0.21–0.75), as well as those supporting more children in the household (OR 0.85, CI 0.72–0.99). Each additional hour of travel time to Lilongwe was associated with a lower GEM score (OR 0.77, CI 0.67–0.88).

### Healthcare access and utilization

**Fig. 1:**
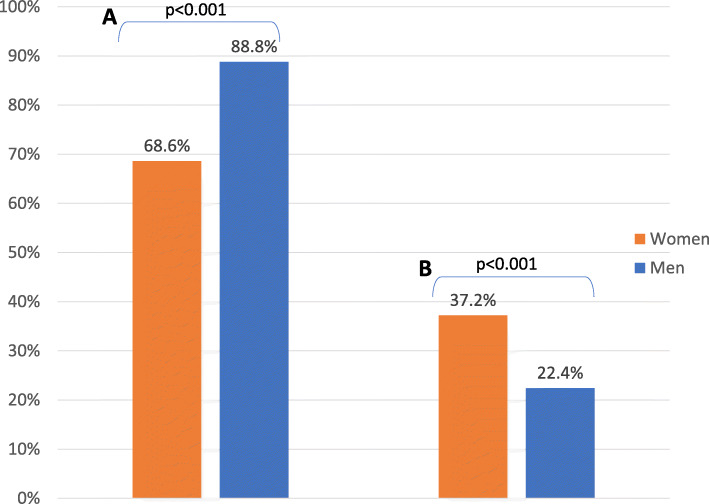
Rates of access to and underutilization of healthcare in Lilongwe, Malawi, June–August 2017, **a** Gender differences in response to, “If you could not afford care, could you get financial support from your family or community? **b** Gender differences in response to, “Have you ever been seriously ill and chosen not to seek care?”

The ability to secure additional funds for healthcare costs from family members or the community was reported by 78.5% of the entire survey population. Women reported a significantly lower ability to secure additional funds (68.6% vs 88.8%, *p* < 0.001) (Fig. 1a). Additional funds were defined as the surplus cost to acquiring care that is not included in the free services offered by the Malawian healthcare system. In regression analysis, being a woman was independently associated with an inability to secure additional funds for healthcare from external sources (OR 0.19, CI 0.08–0.44).

Overall, 30% of respondents reported they had been seriously ill in the past and not sought care. Women were significantly more likely to report underutilization of healthcare than men (37.2% vs. 22.4%, *p* = 0.02) (Fig. 1b) and, being a woman was found to be an independent predictor of underutilization (OR 2.79, CI 1.41–5.53).

### Prioritization in households

**Fig. 2:**
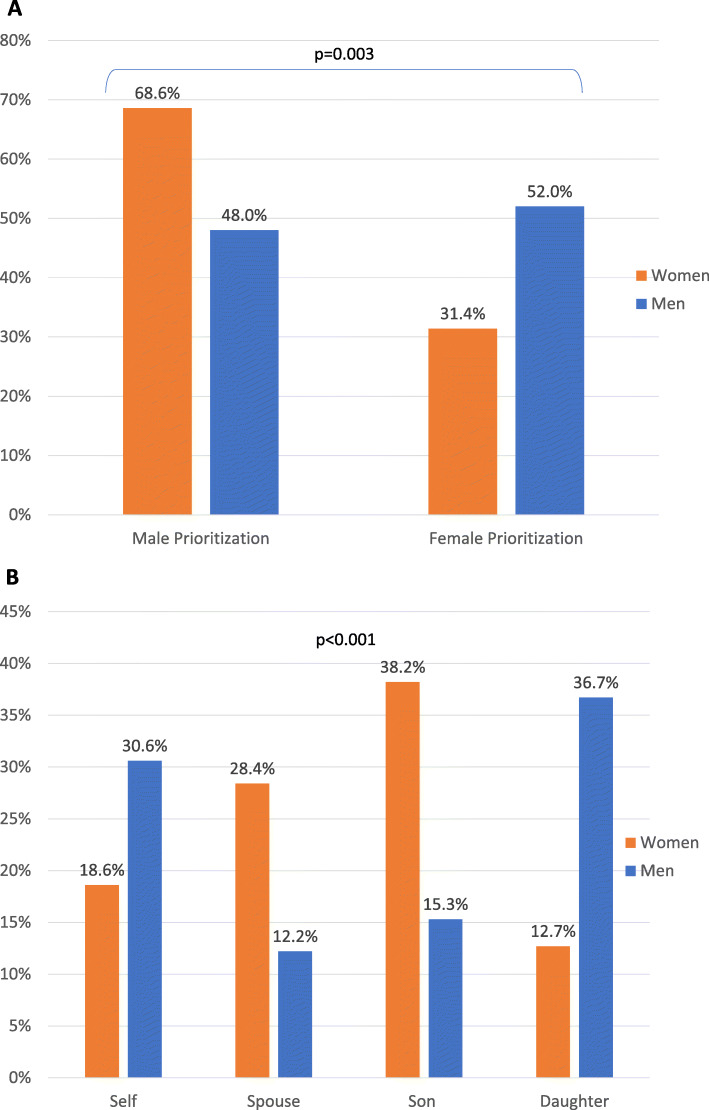
**a** Distribution of Prioritization for Medical Treatment by Gender Prioritization in Lilongwe, Malawi, June–August 2017, **b** Distribution of Prioritization for Medical Treatment by Household Member in Lilongwe, Malawi, June–August 2017. Legend: Men (blue), Women (orange)

Women reported that they would prioritize feeding their sons (63.7%) before others in the household during times of scarcity, whereas men reported equal rates between their sons (38.8%) and daughters (40.8%). When asked about prioritization of household members for healthcare, the most common choice was a man (58.5%) and, more specifically, a son (27.0%) for both genders. Women were more likely to prioritize men for medical treatment (68.6%) whereas men were more equitable (48.0% prioritized men, *p* = 0.003) (Fig. 2a). Women reported prioritizing their sons (38.2%) and husbands (28.4%) over themselves (18.6%) and their daughters (12.7%). Men reported prioritizing their daughters (36.7%) and themselves (30.6%) over their sons (15.3%) and wives (12.2%) (*p* < 0.001) (Fig. 2b).

In multivariate regression analysis, being a woman was associated with a lower likelihood of prioritizing a woman or girl (OR 0.55, CI 0.31–0.99) for food in times of scarcity, as was self-reported Christian faith (OR 0.47, CI 0.23–0.98). Women were also less likely to prioritize themselves or other women or girls for medical treatment (OR 0.41, CI 0.21–0.78). The most common ethnic group in Lilongwe, the Chewa, were more likely to prioritize women for medical treatment compared to other tribal groups (OR 3.87, CI 1.83–8.18). Prioritization of women for education was associated with significantly higher odds of prioritizing women for healthcare (OR 4.13, CI 2.13–8.01), whereas having a woman as the decision-maker for large purchases decreased the odds of prioritizing women for medical treatment (OR 0.38, CI 0.15–0.93) (Table 3).

**Table 3:** Multivariate Logistic Regression for Predictors of Female Prioritization for Medical Treatment in Lilongwe, Malawi, June–August 2017

	Odds Ratio (95% CI)	*P*-value
Female	0.41 (0.21–0.78)	0.007
Chewa Tribal Group	3.87 (1.83–8.18)	< 0.001
Prioritize a Female for Education	4.13 (2.13–8.01)	< 0.001
Female Decision-Maker for Large Purchases	0.38 (0.15–0.93)	0.034

### Decision-making in households

Women were less likely to report themselves as the primary health care decision-maker (47.1% vs. 58.2%, *p* = 0.003), and more likely to report their spouse (45.1% vs. 20.4%, p = 0.003) compared to men. Nearly four in five (79.0%) respondents reported that women were the primary health care decision-maker for children’s health. Most respondents cited men as the healthcare financial decision-makers (68.5%). Only a third of women (33.3%) reported making their own healthcare payment decisions, compared to the large majority of men (85.7%, *p* < 0.001). Multivariate analysis indicated that married respondents were less likely to identify a woman as a healthcare payment decision-maker (OR 0.40, CI 0.16–0.97). Both men and women reported that the primary decision-maker for small household purchases, such as food and clothing, were of their own respective genders (73.5% vs. 58.8%, p < 0.001). For large household purchases, such as bicycles, electrical appliances, or land, men were most often cited as the primary decision-maker by all respondents (81.0%). Similarly, both men and women reported that the money they earn is controlled by men in their households (72.5%). In multivariate analysis, longer transport time, indicating greater distance from Lilongwe, were associated with greater odds of reporting a woman as the small purchase decision-maker (OR 1.25, CI 1.11–1.42) and a lower likelihood of reporting a woman as the decision-maker for large purchases (OR 0.84, CI 0.72–0.98) or for the use of respondent earnings (OR 0.82, CI 0.72–0.94). Respondents who practiced Christianity were less likely to report a woman as the decision-maker for small purchases relative to other religions (OR 0.42, CI 0.18–0.98).

## Discussion

This study sought to explore the individual and societal beliefs surrounding gender roles and their association with access to health in central Malawi. We found that females, as patients, are underrepresented in the KCH census and that women in this region experience greater barriers to healthcare compared to men. Our analyses indicate that women significantly contribute to their own subordinate position in society based on traditional beliefs about their perceived limited utility – beliefs shared by men in this society. The most significant positive predictors of prioritization of women for medical treatment included being from the Chewa tribal group, a matrilineal society, and prioritization of women for education. These results suggest that the influence of inequitable gender norms on health disparities may be attenuated as a woman’s value, empowerment, and health is optimized, as observed in prior studies [35].

In addition to the sex disparity observed in the KCH census, women reported a greater inability to secure additional funds from family or community members to offset their healthcare costs, as well as less healthcare utilization compared to men. In a 2016 study of barriers to surgical care in Cameroon, female sex and an inability to gather additional funds independently increased the odds of a patient’s decision to decline elective surgery [36]. This finding draws a direct line between sociocultural and economic barriers to care that disproportionately affect women and produce the disparities observed in this study population and, likely, communities worldwide.

Use of the GEM scale in Brazil, Chile, Croatia, India, Mexico, and Rwanda have found that more equitable perspectives, such as equal distribution of responsibilities in family, career, social, and education life, may be protective against gender disparities in healthcare [37]. We found that most men and women in our study population held beliefs that a woman’s role is in the home and that men should be the primary decision-maker and work outside the home as represented by low GEM scores. Gender-based health differences can be traced back to these underlying attitudes because they reinforce the subordination of women with regards to social position, economic power, and access to resources [24, 25, 38]. This is supported by a prior study at KCH that analyzed sex disparities in access to emergent surgical care [16]. Another study by Forrester et al. found that females not only presented less frequently for gender neutral peritonitis, but also experienced more delays and longer hospitalizations that men [17]. The authors suggested that sociocultural norms may be one of many reasons that explain the sex disparity and may also explain the skewed gender ratio observed in the adult and pediatric admission rates in this study. Moreover, the disproportionate lack of access to healthcare reported by women in this study is most likely explained in part by these gendered attitudes [21].

A novel finding of this study is that women scored significantly lower than men on the GEM scale, suggesting that women significantly contribute to the propagation of traditional gender roles, and thus may contribute to the subordination of themselves and other women or girls. This self-reported lack of value may explain the pattern we observed wherein women prioritize their husbands and sons over themselves and their daughters for healthcare utilization. These results, in conjunction with the findings that women were the primary health care decision-makers for their children and prioritize their sons in times of food scarcity, confirms a neglect of girls observed before in the literature [27]. Cultural preferences for men and boys has been shown to contribute to higher mortality and morbidity for women and girls in LMICs. This has been attributed to gender norms that support boys’ education, work outside of the home, and thus value, compared to girls [39–41]. Strong son preference by women, combined with their healthcare decision-making role, may be another contributing factor to the sex disparity observed at KCH. Prior studies have described gender equity as a predictor for child health with demonstrated benefits in health and development outcomes for children of both genders [4, 42]. Therefore, our research suggests that women contribute to and are negatively impacted by traditional gender attitudes at all stages of life.

We also found that increased number of children and longer travel time to Lilongwe, indicating rural dwelling, were independently associated with traditional gender attitudes. Those raising more children were more likely to conform to traditional norms, which may indicate a connection between gender perspectives and economic limitations within households. Similarly, the 2008 Understanding Women’s Empowerment report demonstrated that greater wealth is associated with gender-egalitarian beliefs [35]. They found that wealthier women in Malawi were less likely to agree with wife beating for reasons such as child neglect, refusing sex, or burning food. In our study, respondents with longer transport times to urban Lilongwe were more likely to have women as decision-makers for small purchases, men as decision-makers for large economic decisions, and were less likely to have a woman make decisions about the money they earned. Together, these findings confirm our observation that longer transport times and distance from an urban center are associated with low GEM scores and more traditional gender attitudes. This novel finding of the rurality effect on gender attitudes adds an important geographical dimension for consideration in future studies [43].

Male-dominated healthcare and economic decision-making are widely accepted as indicators of women’s disempowerment that obstruct a woman’s ability to advocate for her own health [44–46]. This was also an observation in our study as respondents who were married were less likely to have a female decision-maker for healthcare payments indicating gender inequity. Those who practiced Christianity were less also likely to prioritize a female in times of food scarcity and have a female decision-maker for small purchases. Religion and marriage as predictors of gender equity in this study provides further evidence for the use of sociocultural factors in future studies to holistically evaluate gender disparities.

Gender disparities in heath are heavily influenced by individual and societal attitudes that undervalue women. Our study found that female education and economic empowerment alone is unlikely to change perspectives and practices that are deeply engrained. Our data is consistent with prior studies documenting gender disparities in access to healthcare [15, 35, 47]. The evidence from this study adds to an emerging body of data confirming the need for more large-scale, longitudinal research that evaluates the impact of these factors on healthcare outcomes. Future studies should employ an integrated, life-course approach with culturally relevant measures of women’s health because gender norms are dependent on the culture of the community and likely vary widely. Furthermore, interventions that focus on a woman’s sense of importance and prioritization of their health and the health of their daughters is essential to addressing these barriers to care [48]. In order to alleviate the burden faced by women in LMICs, gender equity is crucial to fully engage women in society.

## Study limitations

Although every effort was made to minimize bias in the study, there are limitations to our analysis. While an ideal study design would have utilized a clustered, community-based survey distribution, due to limited resources, our study cohort was a stratified, systematic sample of respondents at KCH and surrounding markets, included to mitigate selection bias, thus limiting the generalizability of our findings to other LMIC communities. Another potential limitation was the use of self-reported data. Respondents may have answered in a manner they believed was socially acceptable to the data clerks and researcher, potentially resulting in an underestimation of gender disparities.

## Conclusions

To summarize, in Lilongwe, Malawi, we found that gender disparities in heath are heavily influenced by individual and societal attitudes that undervalue women and prioritize men’s health. Targeted interventions are needed to bolster the self-worth of women in these settings to prioritize her health and the health of her daughters. These interventions will help close the gap in access to care that disproportionately affects women. Furthermore, future studies that incorporate gender norms into their design can be used identify, plan, and implement interventions to raise awareness of gender norms and empower women to advocate for their own health, as well as the health of other women and girls.

## Supplementary Information

**Additional file 1:** **Supplementary Table A.** Sociodemographic Characteristics of Respondents by Location in Lilongwe, Malawi, June–August 2017.

## Data Availability

The datasets used and/or analyzed for the study are available from the corresponding author on reasonable request.

## Abbreviations

LMIC: Low- and Middle-Income Country
KCH: Kamuzu Central Hospital, Lilongwe, Malawi
GEM: Gender-Equitable Men Scale
M:F: Male to Female Ratio
OR: Odds Ratio
CI: Confidence Interval
WHO: World Health Organization
