# Publisher Correction to: The gender gap and healthcare: associations between gender roles and factors affecting healthcare access in Central Malawi, June–August 2017

**DOI:** 10.1186/s13690-021-00538-y

**Published:** 2021-02-12

**Authors:** Amee D. Azad, Anthony G. Charles, Qian Ding, Amber W. Trickey, Sherry M. Wren

**Affiliations:** 1 Stanford University School of Medicine, 291 Campus Drive, Stanford, CA 94305 USA; 2 University of North Carolina Department of Surgery, Chapel Hill, NC USA; 3 Stanford-Surgery Policy Improvement Research & Education Center, Stanford, CA USA; 4 Palo Alto Veterans Healthcare System, Palo Alto, CA USA

**Correction to: Arch Public Health 78, 119 (2020)**

**https://doi.org/10.1186/s13690-020-00497-w**

The original publication [1] of this article a part of Fig. 2 was accidentally removed during the publication process. In this correction article the correct (Fig. 1) and incorrect (Fig. 2) version of Fig. 2 are published for reference. The original publication has been updated.

**Fig. 1:**
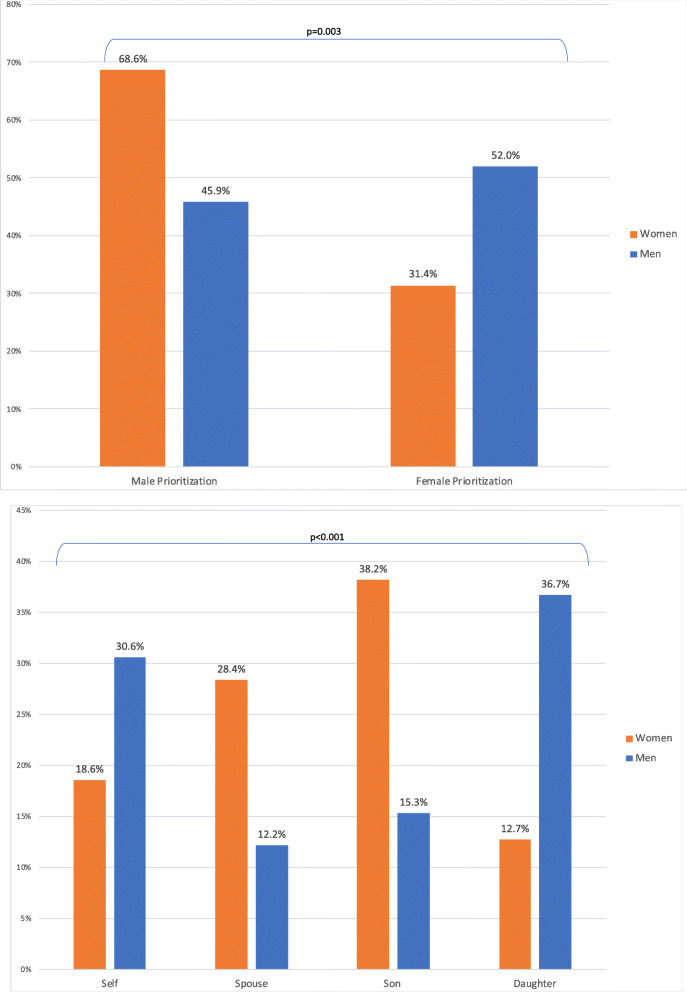
Rates of access to and underutilization of healthcare in Lilongwe, Malawi, June–August 2017, **a** Gender differences in response to, “If you could not afford care, could you get financial support from your family or community? **b** Gender differences in response to, “Have you ever been seriously ill and chosen not to seek care?”

**Fig. 2:**
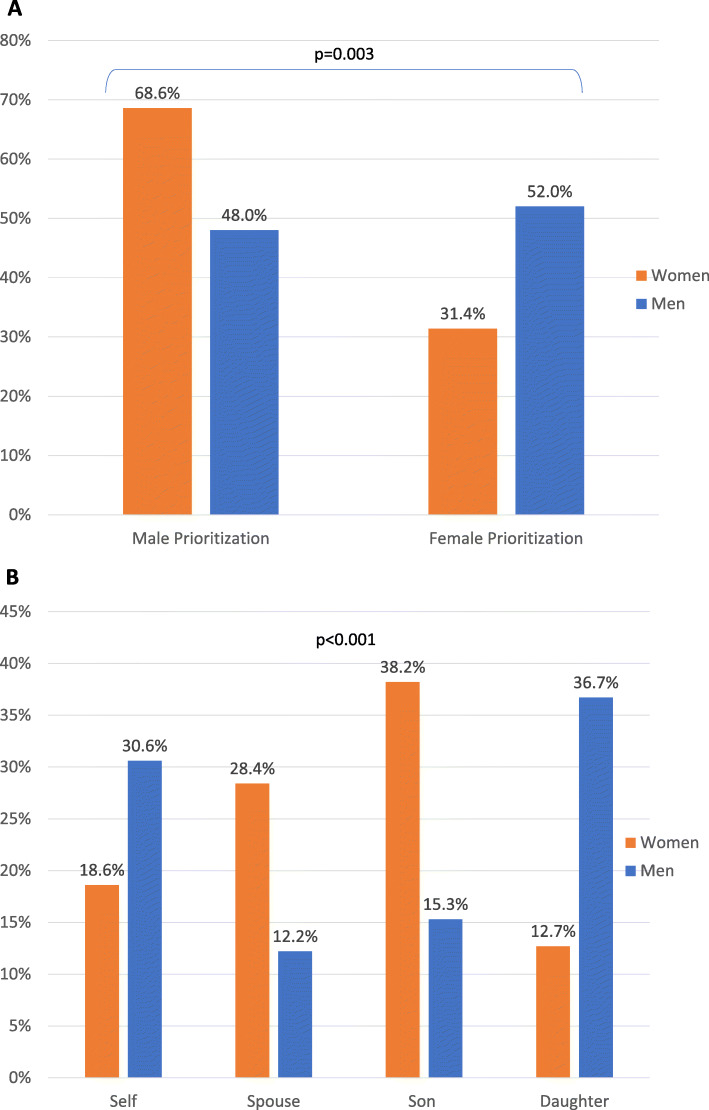
**a** Distribution of Prioritization for Medical Treatment by Gender Prioritization in Lilongwe, Malawi, June–August 2017, **b** Distribution of Prioritization for Medical Treatment by Household Member in Lilongwe, Malawi, June–August 2017. Legend: Men (blue), Women (orange)

Figure 1 the correct version of Fig. 2.

Figure 2 the incorrect version of Fig. 2.

## References

[1] Azad AD, Charles AG, Ding Q (2020). The gender gap and healthcare: associations between gender roles and factors affecting healthcare access in Central Malawi, June–August 2017. Arch Public Health.

