# Supplementary material for: The gender gap and healthcare: associations between gender roles and factors affecting healthcare access in Central Malawi, June–August 2017
Source: Arch Public Health. 2020 Nov 17;78:119. doi: 10.1186/s13690-020-00497-w (PMC7672876; doi:10.1186/s13690-020-00497-w)
Supplement: Supplementary file 1 — Additional file 1: Supplementary Table A. Sociodemographic Characteristics of Respondents by Location in Lilongwe, Malawi, June–August 2017. [file 13690_2020_497_MOESM1_ESM.docx]

| **Supplemental Table A: Sociodemographic Characteristics of Participants by Location in Lilongwe, Malawi, June-August 2017** | | | | |
| --- | --- | --- | --- | --- |
|  | **Total (N=200)** | **Market (N=40)** | **Hospital (N=160)** | P-value* |
| **Gender, n (%)** |  | 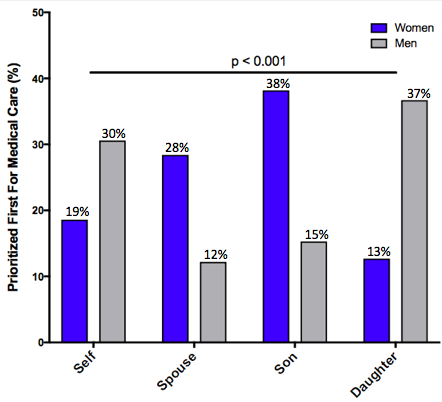 |  | 0.70 |
| Women | 102 (51.0) | 22 (55.0) | 80 (50.0) |  |
| Men | 98 (49.0) | 18 (45.0) | 80 (50.0) |  |
| **Region, n (%)** |  | 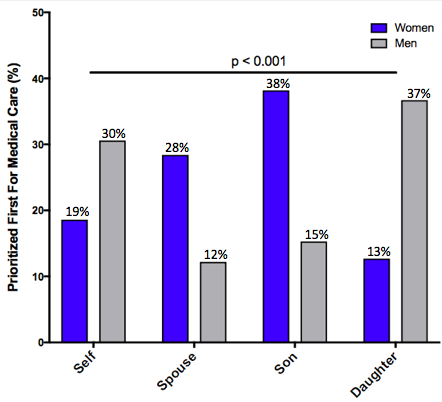 | 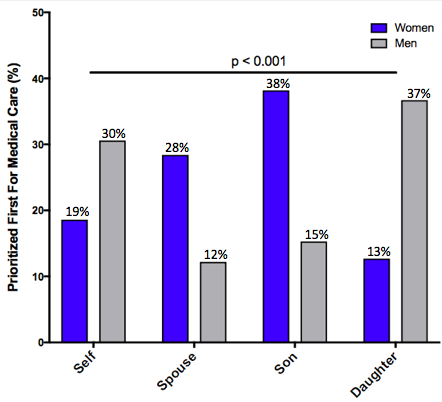 | 0.48 |
| Central Malawi | 176 (88.0) | 37 (92.5) | 139 (86.9) |  |
| Other | 24 (22.0) | 3 (7.5) | 21 (13.1) |  |
| **Tribal Group, n (%)** |  |  |  | <0.001 |
| Chewa | 137 (68.5) | 37 (92.5) | 100 (62.5) |  |
| Ngoni | 38 (19.0) | 0 (0.0) | 38 (23.8) |  |
| Other | 25 (12.5) | 3 (7.5) | 22 (13.8) |  |
| **Religion, n (%)** |  |  |  | 0.45 |
| Protestant | 117 (58.5) | 29 (72.5) | 88 (55.0) |  |
| Roman Catholicism | 46 (23.0) | 6 (15.0) | 40 (25.0) |  |
| Traditional Tribal | 25 (12.5) | 4 (10.0) | 21 (13.1) |  |
| Islam | 9 (4.5) | 1 (2.5) | 8 (5.0) |  |
| Other | 3 (1.5) | 0 (0.0) | 3 (1.9) |  |
| **Marital Status, n (%)** |  |  |  | 0.04 |
| Married | 165 (82.5) | 31 (77.5) | 134 (83.8) |  |
| Separated | 13 (6.5) | 6 (15.0) | 7 (4.4) |  |
| Widowed | 10 (5.0) | 0 (0.0) | 10 (6.3) |  |
| Single | 12 (6.0) | 3 (7.5) | 9 (5.6) |  |
| **Employment Status, n (%)** |  |  |  | 0.31 |
| Self-employed/Business | 178 (89.0) | 40 (100.0) | 138 (86.3) |  |
| Employed | 12 (6.0) | 0 (0.0) | 12 (7.5) |  |
| Unemployed | 2 (1.0) | 0 (0.0) | 2 (1.3) |  |
| Never Worked | 6 (3.0) | 0 (0.0) | 6 (3.8) |  |
| Other | 2 (1.0) | 0 (0.0) | 2 (1.3) |  |
| **Education Level, n (%)** |  |  |  | 0.93 |
| None | 22 (11.0) | 4 (10.0) | 18 (11.3) |  |
| Primary School | 123 (61.5) | 25 (62.5) | 98 (61.3) |  |
| Secondary School | 52 (26.0) | 11 (27.5) | 41 (25.6) |  |
| Higher Education | 3 (1.5) | 0 (0.0) | 3 (1.9) |  |
| **Spouse Employment, n (%)** |  |  |  | 0.004 |
| Self-employed/Business | 148 (89.2) | 25 (62.5) | 123 (76.9) |  |
| Employed | 9 (5.4) | 1 (2.5) | 8 (5.0) |  |
| Unemployed | 4 (2.4) | 1 (2.5) | 3 (1.9) |  |
| Never Worked | 4 (2.4) | 4 (10.0) | 0 (0.0) |  |
| Other | 1 (0.6) | 0 (0.0) | 1 (0.6) |  |
| **Spouse Education Level, n (%)** |  |  |  | 0.10 |
| None | 16 (9.6) | 5 (16.1) | 11 (8.1) |  |
| Primary School | 115 (69.3) | 18 (58.1) | 97 (71.9) |  |
| Secondary School | 34 (20.5) | 7 (22.6) | 27 (20.0) |  |
| Higher Education | 1 (0.6) | 1 (3.2) | 0 (0.0) |  |
| **Age, year, Mean (SD)** | 41.7 (12.1) | 37.2 (11.5) | 42.9 (12.0) | 0.008 |
| **Spouse Age, year, Mean (SD)** | 41.3 (12.4) | 36.9 (12.4) | 42.3 (12.3) | 0.04 |
| **Transport Time, hour, Median (IQR)** | 3.0 (2.0, 6.0) | 2.0 (1.0, 4.3) | 3.25 (2.0, 6.6) | <0.001 |
| **Income (MWK)**, Median (IQR)** | 120000 (60000, 300000) | 180000 (12000, 30000) | 120000 (60000, 300000) | 0.46 |
| **Spouse's Income (MWK)**, Median (IQR)** | 125000 (60000, 300000) | 180000 (45000, 315000) | 120000 (60000, 300000) | 0.28 |
| **Number of Children, Mean (SD)** | 4.1 (2.1) | 3.6 (1.7) | 4.2 (2.2) | 0.06 |
| **Number in Household, Mean (SD)** | 5.7 (2.0) | 5.2 (2.1) | 5.8 (1.9) | 0.12 |
| **GEM Raw Score, Mean (SD)** | 17.2 (2.9) | 17.7 (3.2) | 17.1 (2.8) | 0.3 |
| **GEM Score Level, n (%)** |  |  |  | 0.22 |
| High | 71 (35.5) | 18 (45.0) | 53 (33.1) |  |
| Low | 129 (64.5) | 22 (55.0) | 107 (66.9) |  |
| Missing values: Spouse Employment=34, Spouse Education=34. | | |  |  |
| *P-values were calculated by chi-square test, Fisher’s exact test, independent sample t-test, or Wilcoxon-Mann-Whitney test. | | | | |
| **MWK indicates Malawian kwacha (1 US Dollar = 765.29 MWK as of July16, 2019) | | | |  |
